# *Burkholderia* species in human infections in Mexico: Identification of *B. cepacia, B. contaminans, B. multivorans, B. vietnamiensis,B. pseudomallei* and a new *Burkholderia* species

**DOI:** 10.1371/journal.pntd.0009541

**Published:** 2021-06-29

**Authors:** Georgina Meza-Radilla, Violeta Larios-Serrato, Rigoberto Hernández-Castro, J. Antonio Ibarra, Paulina Estrada-de los Santos

**Affiliations:** 1 Instituto Politécnico Nacional, Escuela Nacional de Ciencias Biológicas. Ciudad de México, México; 2 Ecology of Pathogen Agents Department, General Hospital Manuel Gea González, Mexico City, Mexico; National University of Singapore, SINGAPORE

## Abstract

**Background:**

*Burkholderia sensu stricto* is comprised mainly of opportunistic pathogens. This group is widely distributed in the environment but is especially important in clinical settings. In Mexico, few species have been correctly identified among patients, most often *B*. *cepacia* is described.

**Methodology/Principal findings:**

In this study, approximately 90 strains identified as *B*. *cepacia* with the VITEK2 system were isolated from two medical centers in Mexico City and analyzed by MLSA, BOX-PCR and genome analysis. The initial identification of *B*. *cepacia* was confirmed for many strains, but *B*. *contaminans*, *B*. *multivorans* and *B*. *vietnamiensis* were also identified among clinical strains for the first time in hospitals in Mexico. Additionally, the presence of *B*. *pseudomallei* was confirmed, and a novel species within the *B*. *cepacia* complex was documented. Several strains misidentified as *B*. *cepacia* actually belong to the genera *Pseudomonas*, *Stenotrophomonas* and *Providencia*.

**Conclusions/Significance:**

The presence of different *Burkholderia* species in Mexico was confirmed. Correct identification of *Burkholderia* species is important to provide accurate treatment for immunosuppressed patients.

## Author summary

*Burkholderia sensu stricto* species are opportunistic pathogens in immunosuppressed patients. Both *B*. *cepacia* complex and *B*. *pseudomallei* are widely known around the world but not in Mexico. The identification of these bacteria can be difficult due to the similarity among the species. Tools like MLSA and genome analysis are currently used for identification. Approximately 90 strains identified as *B*. *cepacia* with the VITEK2 system were obtained from two hospitals in Mexico City. The strains were analyzed by MLSA, BOX-PCR and genome analysis. The analysis confirmed the presence of *B*. *cepacia* and identified for the first time *B*. *multivorans*, *B*. *contaminans* and *B*. *vietnamiensis* in Mexican clinical settings. The presence of *B*. *pseudomallei* was confirmed, and a novel species in the *B*. *cepacia* complex was also found. The correct identification of *Burkholderia* species in Mexico is important for appropriate treatment application.

## Introduction

*Burkholderia sensu stricto* contains the *Burkholderia cepacia* complex (Bcc) and *Burkholderia pseudomallei* group. The Bcc includes 24 validly published bacterial species names (LPSN, List of Prokaryotic names with Standing in Nomenclature, lpsn.dsmz.de) and two additional species not yet validly published, *B*. *paludis* and *B*. *servocepacia* [1,2]. The Bcc are opportunistic pathogens causing pneumonia in patients with cystic fibrosis (CF) and chronic granulomatous disease [3]. The Bcc are also found in soil, sediment, water, rhizosphere, plant nodules, flowers, or seeds [4], all of which can act as a reservoir for potential infections for immunosuppressed patients. The distribution of Bcc is worldwide, and species such as *B*. *cenocepacia* are the most prevalent in CF patients [5]. *B*. *contaminans* is an emerging CF pathogen, having been found in Argentina, Spain, United Kingdom, Portugal and Ireland [6]. In México, few Bcc species, including *B*. *cepacia*, *B*. *cenocepacia* and non-identified Bcc strains at the species level, have been isolated from urinary tract, hemocultures, chronic granulomatous disease lung biopsy, catheter and clinical gel [7,8,9,10,11,12].

*B*. *pseudomallei* is the causative agent of melioidosis, a potentially fatal disease for humans in many tropical and subtropical countries [13,14,15,16]. This bacterium is found in soil and fresh surface water. Cases of melioidosis have been detected in a number of countries [17]. In North America, the highest incidence of melioidosis is predicted in Mexico, with 550 cases per 100,000 people each year [18]. Yet, these events are not reported, and melioidosis has been described in only a few cases [17,19]. *B*. *pseudomallei* has been isolated from pediatric patients with pneumonia in Mexico, but melioidosis was not diagnosed at any time [12]. We recently related environmental *B*. *pseudomallei* to two fatal cases of melioidosis in Sonora, Mexico [20]. Although Sonora is not tropical (located in northwest Mexico), the presence of *B*. *pseudomallei* in stressful conditions has been documented previously [21], including in desert regions [22].

Greater awareness of *Burkholderia* spp. in Mexico and more effective tools to correctly detect these species may help to provide accurate treatment of infections in clinical settings.

## Methods

### Bacterial strains and culture conditions

A group of 65 *Burkholderia* sp. strains was provided by Hospital General Dr. Manuel Gea González (HGMGG). These strains were isolated from different human sources, such as bronchus secretions, expectorations, feces, blood, cervical wounds, cultures from catheters and pharyngeal exudates of patients 1–88 years old between 2009 and 2011 (9 patients from 1 to 8 years old, 5 from 20 to 29 years old, 3 from 34 to 39 years old, 6 from 45 to 50 years old, 6 from 52 to 60 years old, 5 from 62 to 70 years old and 7 from 72 to 88 years old) (Tables 1 and S1). A second group comprised 26 strains isolated in Hospital Infantil de México Federico Gómez (HIMFG) from children with pneumonia and initially identified as *B*. *cepacia* [12]. These strains were included in this study to identify them at the species level (Table 1). The type species *B*. *cepacia* ATCC 25416^T^, *B*. *contaminans* LMG 23361^T^, *B*. *multivorans* LMG 13010^T^, *B*. *vietnamiensis* TVV75^T^ and *B*. *cenocepacia* J2315^T^ and reference strain *B*. *cenocepacia* TAtl-371 were included in the analyses. The strains were regularly kept in LB Petri dishes at room temperature and preserved with 35% glycerol at -70°C.

**Table 1:** *Burkholderia* strain list and BOX pattern isolated from patients at two hospitals in Mexico, City.

BOX pattern	Strains	Isolation source
BOX pattern I *B*. *cepacia*	581, 582, 583, 593, 596, 597, 608, 612, 634, 648, 649, 652, 659, 669, 670, 673, 674, 689, 705, 712, 812, **848**, 887, 893, 894, 899, 907, 928, 939, 961, 6772	Bronchus secretion culture^1^
	888	Hemoculture^1^
	900	Culture from catheter^1^
	924	Expectoration culture^1^
BOX pattern II *B*. *cepacia*	600, 677, **810**, 823, 910, 933	Bronchus secretion culture^1^
BOX pattern III *B*. *cepacia*	594, 599, 614, 650, **921**, 923, 925, 6491, 6742	Bronchus secretion culture^1^
	885	Feces culture^1^
	897	Cervical wound culture^1^
	908	Pharyngeal exudates culture^1^
BOX pattern IV *B*. *cepacia*	598, **871**	Bronchus secretion culture^1^
BOX pattern V *B*. *contaminans*	**40H**	Pharyngeal exudates^2^
BOX pattern VI *B*. *contaminans*	337D, **1H**, 60H	Pharyngeal exudates^2^
BOX pattern VII *B*. *contaminans*	426D, **584U**, **661U**, 923U, BC1608	Pharyngeal exudates^2^
BOX pattern VIII *B*. *contaminans*	**407H**	Pharyngeal exudates^2^
BOX pattern IX *B*. *multivorans*	235H, 236H, **785H**, 467H	Pharyngeal exudates ^2^
BOX pattern X *B*. *pseudomallei*	45H, 205H, **294H**, 297H, 306H, 305H, 339H, 337H	Pharyngeal exudates^2^
BOX pattern XI *B*. *vietnamiensis*	**184D**, 662D	Pharyngeal exudates^2^
BOX pattern XII *Burkholderia* sp.	**500H**, 501H	Pharyngeal exudates^2^

^1^, HGMGG, Hospital General Dr. Manuel Gea González.

^2^, HIMFG, Hospital Infantil de México Federico Gómez. In bold strains chosen for genome sequencing.

### 16S rRNA and *atpD* gene amplification and phylogenetic analysis

Total DNA was isolated by growing the strains in 5 mL LB and incubating overnight at 30°C with shaking (120 rpm). DNA was obtained using the cetyl trimethyl ammonium bromide (CTAB) method according to Moore and Dowhan [23]. The 16S rRNA gene was amplified with primers 27F/1492R [24]. The *atpD* gene was amplified according to Baldwin et al. [25]. The PCR fragments were sequenced at Macrogen Inc. (https://www.macrogen.com) using the same primers for amplification. The sequences were analyzed and assembled with ChromasPro 2.1.5 (Technelysium Pty Ltd). The 16S rRNA gene sequences were analyzed with BlastN and *atpD* gene sequences with BlastX at NCBI (https://blast.ncbi.nlm.nih.gov). The sequences were aligned with Clustal Omega (https://www.ebi.ac.uk/Tools/msa/clustalo/) and the best-fit model was used to analyze the data with jModelTest 2.1.10 [26,27]. Bayesian phylogenetic analysis was inferred with BEAST 2 (www.beast2.org), performed separately for each gene and with the concatenated sequences. The concatenation of 16S rRNA and *atpD* genes was carried out with Mesquite 3.51 (https://www.mesquiteproject.org/). The tree was displayed with FigTree v1.4.4 (http://tree.bio.ed.ac.uk/software/figtree/), and the Bayesian posterior probability was included in each node of the phylogenetic tree.

### Polymerase chain reaction amplification of the BOX dispersed-repeated motif (BOX-PCR) analysis

All strains were analyzed with BOX-PCR using the BOXA1R primer [28]. Cycling conditions were 95°C for 5 min and then 35 cycles of 95°C for 1 min, 63°C for 1 min and 72°C for 6 min and a final elongation cycle of 10 min at 72°C. The BOX patterns were observed in 1% agarose gels.

### Whole-genome sequencing and analysis

Selected strains were grown in 40 mL LB and incubated overnight at 30°C. The total DNA was isolated using the method of Moore and Dowhan [23]. The genome sequence was obtained at Novogene (https://en.novogene.com/) using Illumina Platform PE150 with a 350-bp insert DNA library. For the genome assembly, the quality control analysis was carried out with FastQC to 0.11.9 (https://www.bioinformatics.babraham.ac.uk/projects/fastqc/), checking for the presence of adapters and length of reads. The clipping and filtering of the information was performed with Trimmomatiq 0.39 [29], keeping only those sequences with phred quality greater than 28. All samples were assembled with an optimized method of on de Bruijn graphs and automatic adjustment of the K value with the SPAdes 3.14 program [30]. Some of the assemblies underwent a cleaning process to remove spurious sequences using Blast 2.9.0 [31] comparing to a *Burkholderia* genome bank. Metrics such as N50 and misassembles were obtained using QUAST 5.0.2 [32]. Annotation was performed using the standard operating procedure at DOE-JGI Microbial Genome Annotation Pipeline (MGAP v.4). Average Nucleotide Identity (ANI) calculations were obtained with JSpeciesWS [33], using ANIm (based on the MUMmer algorithm). The digital DNA-DNA hybridization (dDDH) was calculated in silico with the Genome-to-Genome Distance Calculator (GGDC 2.1) using the BLAST method [34]. Results were based on recommended formula 2 (identities/HSP length), which is independent of genome length and appropriate for incomplete genomes.

### Phylogenomic analysis

A phylogenomic analysis was carried out to determine the relatedness of *Burkholderia* strains with *Burkholderia* species using the program up-to-date bacterial core gene (UBCG), which is based in the phylogenetic analysis of 92 core genes, that were previously selected from the analysis of 1429 species available at EzBioCloud (https://www.ezbiocloud.net/), representing 28 phyla, including *Burkholderia* species [35]. Single-nucleotide polymorphism (SNPs) was analyzed with strain 294H and compared to other *B*. *pseudomallei* strains. The analysis was performed with maximum parsimony based on core SNPs with Parsnp, a component of the Harvest 1.3 software (https://github.com/marbl/harvest).

### Multilocus sequence typing (MLST) analysis

MLST allele sequences were analyzed by BLAST searches using the sequenced genomes with the Bcc and *B*. *pseudomallei* PubMLST Databases [36].

### Antibiotic susceptibility

All strains were tested with the VITEK2 system for antibiotic susceptibility.

## Results

### Bacterial identification and phylogenetic analysis

**Fig 1:**
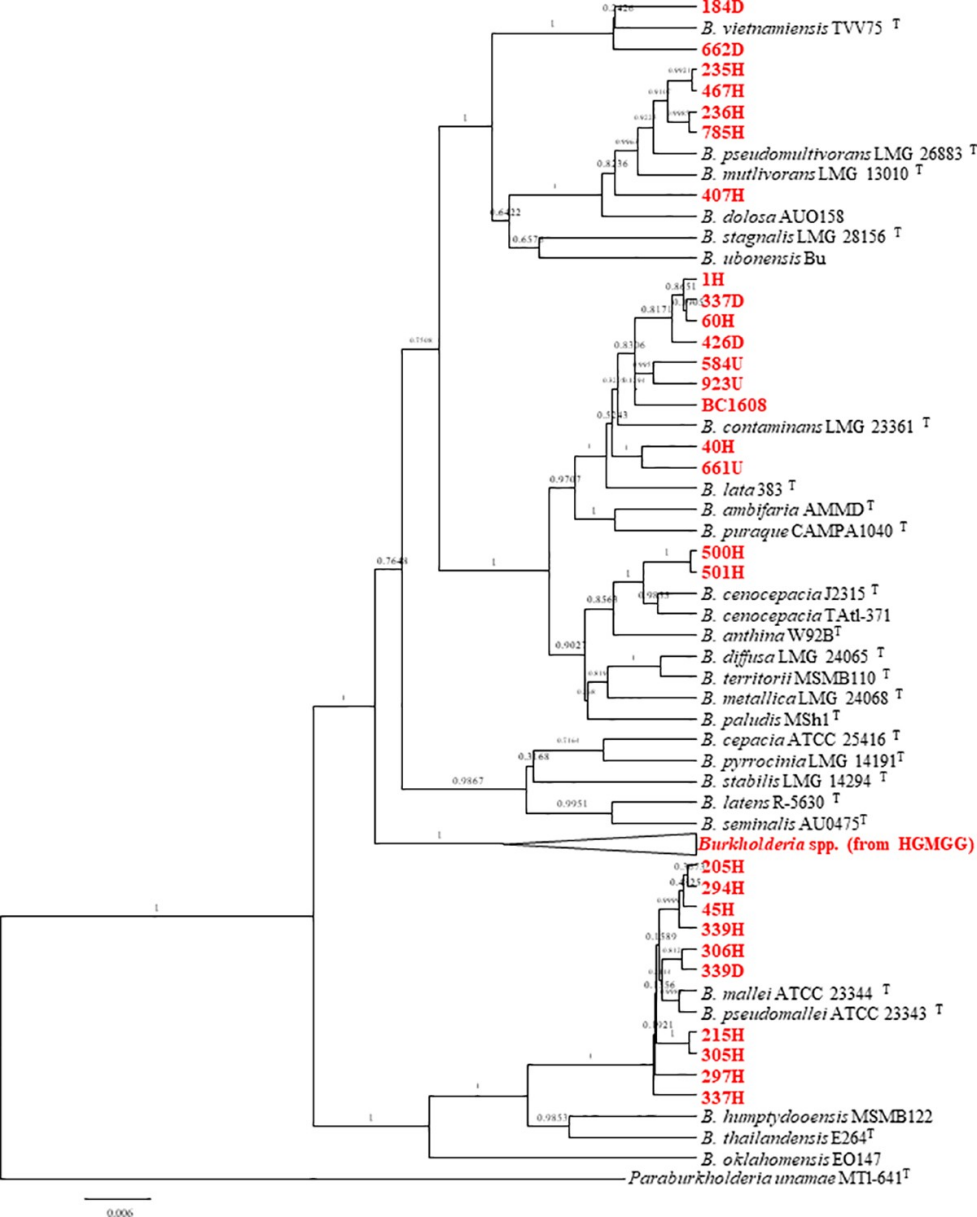
Phylogenetic analysis of *Burkholderia* species with 16S rRNA and *atpD* genes with the Bayesian method and the model GTR+G+I. *Paraburkholderia unamae* MTl-641^T^ was used as an outgroup. Bar, the nucleotide differences between the sequences. Bayesian posterior probabilities values shown in branches.

The 65 strains isolated from multiple patients at the HGMGG were initially identified as *B*. *cepacia* with the VITEK2 system. However, according to the analysis of the 16S gene, only 54 of these strains belonged to the Bcc. The 16S ranges of similarity were 99.6–100% to *B*. *anthina*, 99.42–100% to *B*. *territorii* and 99.34–99.93% to *B*. *cepacia*. By using the same analysis, the rest of the strains (11 of them) were identified as belonging to the genera *Pseudomonas* (3 strains), *Stenotrophomonas* (5 strains), and *Providencia* (3 strains). The selected 26 strains from HIMFG were identified as *B*. *contaminans* (99.69–100%), *B*. *lata* (99.58–100%), *B*. *arboris* (99.64–99.85%), *B*. *vietnamiensis* (99.28–99.98%), *B*. *latens* (99.06–99.71%), *B*. *cenocepacia* (99.06–99.71%), *B*. *puraquae* (99.58–99.85%) and *B*. *pseudomallei/B*. *mallei* (99.93%). The 16S rRNA gene phylogenetic analysis of all *Burkholderia* species (27 currently described *Burkholderia* species), including those from HGMGG and HIMFG, and the type species of *Paraburkholderia unamae* used as an external species, showed that the strains were not clearly grouped with any of the species from the Bcc, despite the high similarity found with the 16S rRNA gene analyses, except for a group from HIMFG that was close to *B*. *pseudomallei/B*. *mallei* strains (S1 Fig). Since the phylogenetic analysis with 16S rRNA genes has low taxonomic resolution, which is in line with several reports [37,38,39], a phylogenetic analysis was performed with *atpD* gene, using the same number of strains. The *atpD* gene was used because it is one of the genes listed in the PubMLST for the taxonomical analysis of Bcc (https://pubmlst.org/organisms/burkholderia-cepacia-complex). Phylogenetic analysis of the *atpD* gene gave a better resolution, showing that the strains from HGMGG formed a large cluster, and the strains from HIMFG clustered in few groups but none clearly close to any *Burkholderia* species (S2 Fig). The concatenation of both 16S rRNA and *atpD* genes to seek a better resolution and identification of the strains, resulted in a large group containing all strains from HGMGG (Fig 1). Strains 184D and 662D from HIMFG were close to *B*. *vietnamiensis*, with similarity of 99.28 and 99.93%, respectively, and 99.2% similar to each other. Other HIMFG strains, such as 500H and 501H, grouped with *B*. *cenocepacia* but the 16S rRNA showed more similarity to *B*. *contaminans* (99.85%). The strains identified as *B*. *pseudomallei/B*. *mallei* were intermingled with these species (Fig 1). The positioning of the rest of the strains from HIMFG was not clear, showing no association to any particular *Burkholderia* species.

### BOX-PCR analysis

Since the resolution in the analysis of 16S rRNA and *atpD* genes was poor in general, the BOX profiles were analyzed visually to determine clonality and to define possible *Burkholderia* subgroups among the isolates. Twelve BOX patterns were found among the HGMGG and HIMG strains (Table 1); as expected, none of these patterns was shared among the strains from both hospitals. Strains collected from HGMGG displayed four different patterns with pattern I present in the largest number of strains (31 strains). Strains from HIMFG displayed eight different patterns, with pattern VIII present in 10 strains. At least one strain with each BOX pattern was selected for genome sequencing to determine the *Burkholderia* species they belonged to. The results suggested that many of the isolates were clonal and that an internal outbreak might have happened at HGMGG.

### Whole-genome analysis

The genomes of 12 selected strains were analyzed with the ANI and dDDH tools, considering that cutoff values of 95–96 and 70%, respectively, as the delineation of a species [40]. The comparative analyses showed that the strains with BOX patterns I, II, III and IV from HGMGG belonged to *B*. *cepacia*, with an ANI value of 97.53–97.60% and a dDDH of 76.50–76.80% (Table 2). The strains obtained from HIMFG with BOX patterns V, VI, VII and VIII were identified as *B*. *contaminans* (ANI 98.68–99.98% and dDDH 83.8–87.50%). The BOX pattern IX was recognized as *B*. *multivorans* (ANI 99.10% and dDDH 91.70%). Those with BOX pattern X were identified as *B*. *pseudomallei* (ANI 99.25% and dDDH 93.7%) and with BOX pattern XI as *B*. *vietnamiensis* (ANI 98.46% and dDDH 90.50%). The strain with the BOX pattern XII (strain 500H) was close to *B*. *cenocepacia*, but, according to the genome comparative analysis, it belongs to “*B*. *servocepacia”* (ANI 97.88% and dDDH 83.00%). However, this bacterial species has not yet been validated, and therefore it is not present in the LPSN.

**Table 2:** Identification of *Burkholderia* strains by genome analyses.

Strain	pattern	Analyses	*B*. *cepacia* ATCC 25416^T^	*B*. *contaminans* LMG 23361^T^	*B*. *multivorans* NCTC 13007^T^	*B*. *pseudomallei* ATCC 23343^T^	*B*. *vietnamiensis* LMG 10929^T^	*B*. *cenocepacia*	
								J2315^T^	TAtl-371
**848**	I	ANI	97.53	93.19	89.43	86.69	87.57	90.18	90.23
		dDDH	76.60	48.40	34.30	26.20	35.40	43.50	43.10
**810**	II	ANI	97.60	93.51	89.52	86.74	87.43	90.00	89.87
		dDDH	76.80	49.90	34.60	26.50	36.20	44.40	44.00
**921**	III	ANI	97.60	93.49	89.52	86.77	87.43	89.97	89.86
		dDDH	76.80	49.80	34.60	26.50	36.10	44.40	44.00
**871**	IV	ANI	97.53	93.18	89.42	86.69	87.57	90.17	90.22
		dDDH	76.50	48.40	34.30	26.20	35.40	43.50	43.00
**40H**	V	ANI	93.42	99.98	89.33	86.68	87.53	90.06	90.12
		dDDH	49.80	83.8	34.30	26.20	35.10	43.60	43.00
**1H**	VI	ANI	93.61	98.75	89.40	86.69	87.28	90.04	89.77
		dDDH	50.60	87.10	34.50	26.50	35.70	44.40	43.50
**584U**	VII	ANI	93.35	98.68	89.30	86.67	87.53	90.21	90.26
		dDDH	49.20	87.50	34.20	26.20	34.80	43.50	42.90
**661U**	VII	ANI	93.35	98.68	89.30	86.68	87.53	90.22	90.27
		dDDH	49.20	87.40	34.20	26.20	34.80	43.50	42.90
**407H**	VIII	ANI	93.35	98.67	89.30	86.68	87.53	90.19	90.25
		dDDH	49.20	87.30	34.20	26.20	34.80	43.50	42.90
**785H**	IX	ANI	89.50	89.35	99.10	86.96	86.65	86.99	86.61
		dDDH	34.80	34.30	91.70	27.00	33.80	35.20	34.60
**294H**	X	ANI	86.76	86.66	86.99	99.25	81.84	81.33	80.99
		dDDH	26.50	26.30	27.20	93.70	27.00	26.90	26.70
**184D**	XI	ANI	87.55	87.48	86.38	81.49	98.46	87.89	87.66
		dDDH	35.80	35.00	33.90	26.80	90.50	36.80	36.1
**500H**	XII	ANI	90.33	90.16	86.45	80.92	86.71	93.90	97.88
		dDDH	43.10	42.60	34.60	26.50	36.10	59.50	83.00

ANI and dDDH values among *Burkholderia cepacia* strains from this study were 99.99–100 and 99.3–100%, respectively. ANI and dDDH among *B*. *contaminans* strains from this study were 98.71–100 and 87.70–100%, respectively. In grey are marked the values that correspond to the identified species.

**Fig 2:**
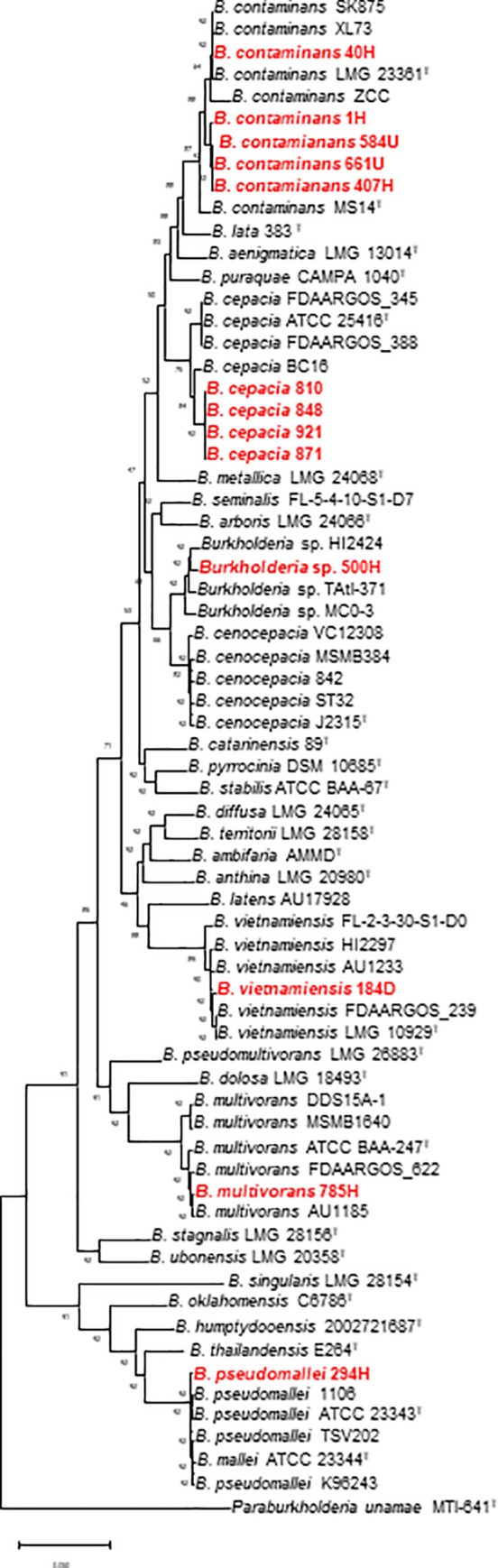
Phylogenetic tree inferred using up-to-date bacterial core genes (concatenated alignment of 92 core genes). A total of 91554 nucleotide positions were used. The phylogenetic tree was inferred with the GTR + CAT model. Gene support indices (GSIs) and percentage bootstrap values are given at branching points. Bar, 0.05 substitutions per position. Bayesian posterior probabilities values shown in branches.

The genome sizes of the clinical strains were in the range of 6.5–8.8 Kbp, which is a genome size expected for bacteria in the *Burkholderia* genus. More genome features are presented in S2 Table. A phylogenomic analysis was performed with concatenating 92 genes with the UBCG pipeline. In this analysis, the type strain and four to five reference strains from each of *B*. *cepacia*, *B*. *cenocepacia*, *B*. *multivorans*, *B*. *contaminans*, *B*. *vietnamiensis*, *B*. *pseudomallei* and *“B*. *servocepacia”* species were included. The reference strains were selected on the basis of sharing more than 70% similarity by dDDH to the type strain to avoid using incorrectly classified species. The topology of the tree showed that all strains analyzed in this study were grouped with the respective species (Fig 2) and the strain 500H was clustered with “*B*. *servocepacia*” strains.

**Fig 3:**
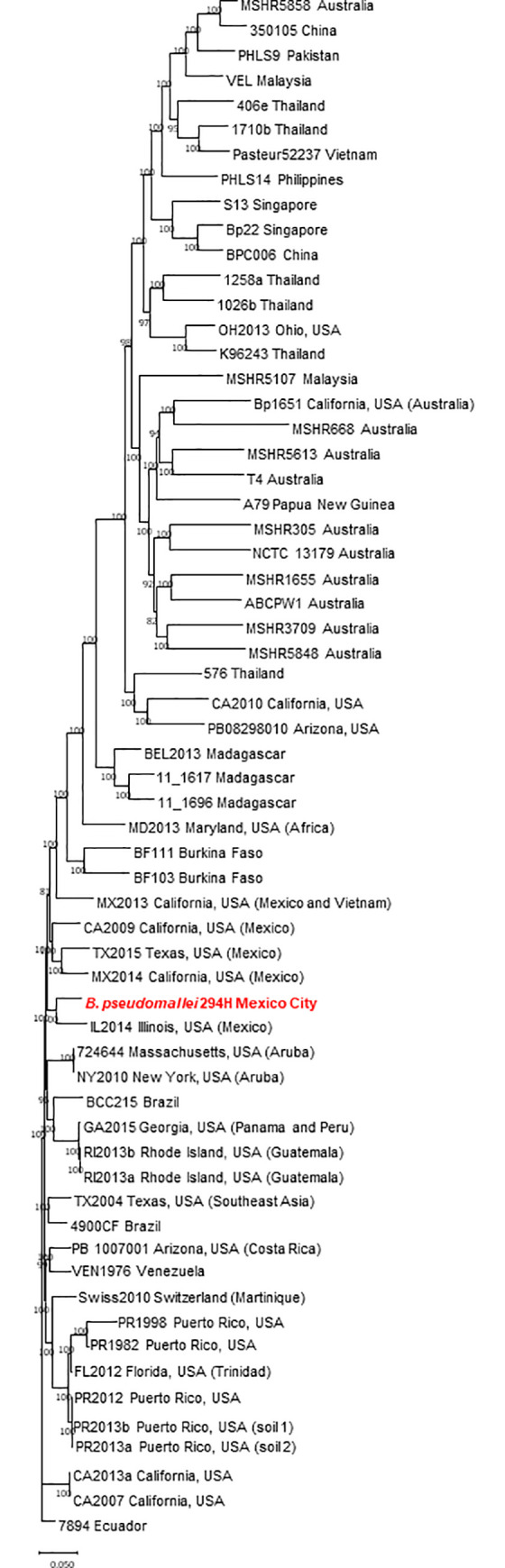
Maximum-parsimony phylogenetic analysis of *Burkholderia pseudomallei* strains based on core single-nucleotide polymorphisms (SNPs) using Parsnp, a component of the Harvest 1.3 software (https://github.com/marbl/harvest). *B*. *pseudomallei* 294H, a clinical strain isolated in Mexico City, is shown in red. The tree was rooted to the most ancestral strain 7894 isolated in Ecuador in 1960. Numbers at each node are bootstrap percentages. Scale bar indicates nucleotide substitutions per SNP.

A dendrogram based on maximum parsimony from analysis of core SNPs for strain 294H was carried out comparing with a number of *B*. *pseudomallei* strains that were isolated from patients with melioidosis living in different locations [41]. Since strain 294H had the same BOX pattern with the rest of the *B*. *pseudomallei* strains analyzed in this study, this strain was randomly selected for genome sequencing and for SNPs analysis. The result showed that strain 294H clustered with *B*. *pseudomallei* strains that were isolated from people living in the US who had a history of travelling to Mexico and other countries in the American continent (Fig 3). Strain 294H was close to a strain isolated from a person with melioidosis living in Illinois, US, who previously had travelled to Mexico. This result shows the presence of the etiologic agent of melioidosis.

### MLST analysis

The strains’ genomes were analyzed in the PubMLST Database to determine whether they correspond to a defined sequence type (ST) or they represented a new ST. The *B*. *cepacia* strains belonged to a single ST, which was ST 9 (S2 Table). The *B*. *contaminans* strains belonged to two established STs, ST 482 and ST 102. The *B*. *vietnamiensis* strain belonged to the known ST 596. However, the *B*. *multivorans* strains studied here represents a novel ST, which was assigned ST 1867. *B*. *pseudomallei* strain 294H also represents a new ST, which was assigned ST 1872 (S2 Table). Since *Burkholderia* sp. 500H represents a novel species, the ST will be established once the species is proposed. These results showed that new STs were identified in some of the strains in this work.

### Antibiotic susceptibility

Analysis with the VITEK2 system showed that all strains were resistant to ticarcillin/clavulanic acid, 87% were sensitive to trimethoprim/sulfamethoxazole, 94% were sensitive to meropenem, 82% were sensitive to levofloxacin and only 28% were sensitive to ceftazidime. These results showed that most of the strains analysed here are susceptible to elective antibiotics, although a small proportion are resistant, underscoring the relevance of defining the antibiotic sensitivity of all *Burkholderia* isolated in clinical settings.

## Discussion

Bcc and *B*. *pseudomallei* are widely distributed in the world, but the evidence of their presence in Mexico is scarce, both in clinical settings and in the environment. Identification of these microorganisms at the species level is very problematic. Species from the Bcc can share up to 100% similarity in 16S rRNA gene sequences [39], prompting to the use of more efficient tools, such as MLSA and ultimately genome sequencing.

Our analysis of *Burkholderia* strains isolated from human infections in two different hospitals in Mexico City showed that the identification system is poor, since all the strains were identified as *B*. *cepacia*, but we found that many belong to other *Burkholderia* species and even other genera. The proper identification of a bacterial species is important to provide the appropriate treatment, which is relevant in *Burkholderia* species as they are highly resistant to many antibiotics. For example, all strains studied were resistant to ticarcillin/clavulanic acid, but most of the strains were sensitive to meropenem. *B*. *pseudomallei* is efficiently treated with ceftazidime, meropenem or trimethoprim/sulfamethoxazole, and the *B*. *pseudomallei* strains studied here were sensitive to these antibiotics as well. Accurate identification of *B*. *pseudomallei* might lead to successful treatment of the patient. Unfortunately, this this did not happen in two fatal cases in northern Mexico; since the melioidosis caused by *B*. *pseudomallei* is barely known in Mexico, correct antibiotic treatment was not provided to the children, who died within a few hours after the admission to a hospital [20].

The analysis of 16S rRNA gene sequence showed poor resolution for the position of the *Burkholderia* strains in the phylogenetic tree. This is expected in this genus and with this gene. But when the *atpD* gene sequence was added to the analysis, the resolution of the bacterial positioning improved significantly, and the strains from HGMGG and HIMFG formed different clusters. Only the strains from HGMGG clustered with *B*. *cepacia*. This species is not typically recognized with highly distribution in clinical settings, as *B*. *cenocepacia* and *B*. *multivorans* are [5].

To group the strains and select some of them for genome sequencing, BOX-PCR was used, revealing 12 BOX patterns. One strain from each profile was selected for genome sequencing. The ANI and dDDH results confirmed that the strains from HGMGG belonged to *B*. *cepacia*, while the strains from HIMFG were identified as *B*. *contaminans*, *B*. *multivorans*, *B*. *vietnamiensis* and *B*. *pseudomallei*. A novel species in the Bcc was also found among the strains from HIMFG. In Mexico, *B*. *cepacia* has been found in several hospitals; however, the identification methodology is not always mentioned, and when automated systems, as used in this study, might not accurately identify the strains [7,11]. Although most of the strains from HGMGG were confirmed as *B*. *cepacia*, other strains had been mistakenly identified as this species, but belonged to other genera, such as *Pseudomonas*, *Stenotrophomonas*, and *Providencia*. *B*. *cenocepacia* and *B*. *multivorans* are Bcc species largely distributed in clinical settings, mainly affecting immunosuppressed patients [42,43,44,45,46,47]. In Mexico, *B*. *multivorans* strains have not been reported in clinical settings; this is the first report describing this species in Mexico. *B*. *contaminans* is considered an emerging CF pathogen in several countries [6], but not in Mexico. *B*. *vietnamiensis* strains have been isolated previously in Mexico, but from the environment. A number of these strains were obtained from maize, coffee and sorghum rhizosphere and rhizoplane in different geographic regions of Mexico [48]. *B*. *vietnamiensis* has been proposed as a plant growth-promoting rhizobacterium (PGPR) [49], but its use in agriculture is ethically incorrect since it is an opportunistic pathogen of the Bcc and has been found in patients with CF [50]. The presence of *B*. *vietnamiensis* in Mexican soils may represent a reservoir for the infection with this microorganism in immunosuppressed patients. Scientific and agricultural communities around the world are encouraged to stop proposing the use of Bcc strains as a PGPRs, as they are opportunistic pathogens.

By using genome sequencing, we corroborated the identification of strain 294H as *B*. *pseudomallei* [12]. This species is not well known in Mexico as few cases have been detected since 1958 [17]. The disease produced by *B*. *pseudomallei*, melioidosis, is present in Mexico, but it is a neglected disease because it is not accurately identified and reported. Recent cases with fatal outcomes [19,20], suggest that greater awareness of this bacterium and the disease is needed to prepare the health care staff to deal with the disease. Since the distribution of *B*. *pseudomallei* is worldwide, an increasing interest has emerged in the origin of *B*. *pseudomallei* strains. Several methods have been used to explore this origin, such as MLST and SNPs [38]. Some studies using SNPs have shown the association of clinical *B*. *pseudomallei* strains with environmental isolates, establishing an epidemiological link to understand the source of infection [51,52,53]. Our analysis with SNPs showed that strain 294H grouped with a strain that was isolated from a melioidosis patient in the US (Illinois) who had travelled to Mexico. Originally, the strain IL2014 formed a group with strains CA2009, MX2013, MX2014 and TX2015, all associated with clinical cases in patients residing in or visited Mexico [41]. However, strain 294H split this group and clustered closely with IL2014. This result show that more studies about the isolation of more *B*. *pseudomallei* strains are necessary to understand the origin of melioidosis infections both in Mexico but also in the US, since many US citizens travel for pleasure or work to Mexico. Moreover, since the US seems not endemic of melioidosis and many strains were isolated from people travelling to Mexico [41], our country might be endemic for *B*. *pseudomallei*.

All *Burkholderia* genomes were analyzed in the MLST database to determine whether they belonged to known STs or represented new ones. This database includes more than 100 different microbial species and genera, and it is intended to address several functional questions, such as activities of different variants that lead to key phenotypes. The Bcc PubMLST database functions to study the epidemiology and diversity of Bcc species. The *B*. *cepacia* strains from this study belonged to a single type, which was ST 9. This ST does not seem to be widely distributed since the database contains only two strains identified as ST 9: one isolated in Canada in 1997 (abdomen from non-CF patient) and a second isolated in Spain in 2009 (sputum from a CF-patient). The strains identified as *B*. *contaminans* were placed within ST 102 and ST 482. These STs are better represented, including 39 strains and 23 strains, respectively. The strains from *B*. *multivorans* and *B*. *pseudomallei* represented new STs; the former was assigned ST 1867 and the latter as ST 1872. However, the number of STs might be underestimated since not all Bcc species are present in the PubMLST database and the distribution of Bcc in the environment and clinical setting is poorly known in Mexico. However, we believe that the Mexican ST’s might be endemic of Mexico, according to our previous analysis [54], but more studies should be carried out.

Additionally, two strains close to *B*. *cenocepacia* were found. By analyzing the genome of strain 500H, which shared the same BOX pattern with strain 501H, we found that it belonged to the novel species “*B*. *servocepacia”* [2]. The proposal of “*B*. *servocepacia”* was based on a comparison among many *B*. *cenocepacia* strains. The result of this analysis showed that *B*. *cenocepacia* could be divided in two major groups, and a few other strains might still represent novel species. Therefore, “*B*. *servocepacia”* was proposed and strain TAtl-371 was selected as the type strains. However, the species name of “*B*. *servocepacia”* has not been validated following the rules of the International Code of Nomenclature of Prokaryotes [55] and is not listed at the LPSN. Thus, we decided to maintain the name *Burkholderia* sp.

According to the identification of the strains, and taking into account the hospital of origin, the isolation source of the strains and the age of the person from whom the strains were isolated, it seems that no correlation exists. Only *B*. *cepacia* was identified from HGMGG but different species were obtained from HIMFG. The range of age was different in the patients from each hospital, but the isolation source might be important since in HGMGG most of the strains were isolated from bronchus secretions and in HIMFG from pharyngeal exudates.

Considering these results, the potential diversity of *Burkholderia* species in Mexico is not yet well understood. The distribution of species from the Bcc in Mexico is barely known, both in clinical settings and in the environment. The latter is important as a reservoir for potential infection in immunosuppressed patients which could explain the presence of Bcc and *B*. *pseudomallei* in human infections. The correct identification of *Burkholderia* species might help to provide accurate treatment for immunosuppressed patients.

## Supporting information

S1 Fig Phylogenetic analysis of 16S rRNA gene sequences from *Burkholderia* species. The analysis was performed with the Bayesian method. Bar, the nucleotide differences between the sequences. Bayesian posterior probabilities values shown in branches.
(TIF)

S2 Fig Phylogenetic analysis of *atpD* gene sequences from *Burkholderia* species. The analysis was performed with the Bayesian method. Bar, the nucleotide differences between the sequences. Bayesian posterior probabilities values shown in branches.
(TIF)

S1 Table *Burkholderia* strain list isolated from patients with different ages at two hospitals in Mexico, City. (DOCX)

S2 Table Genomic features of *Burkholderia* strains isolated from human infections in Mexico. (DOCX)
