# Supplementary figures and images for: Burkholderia species in human infections in Mexico: Identification of B. cepacia, B. contaminans, B. multivorans, B. vietnamiensis,B. pseudomallei and a new Burkholderia species
Source: PLoS Negl Trop Dis. 2021 Jun 29;15(6):e0009541. doi: 10.1371/journal.pntd.0009541 (PMC8274841; doi:10.1371/journal.pntd.0009541)

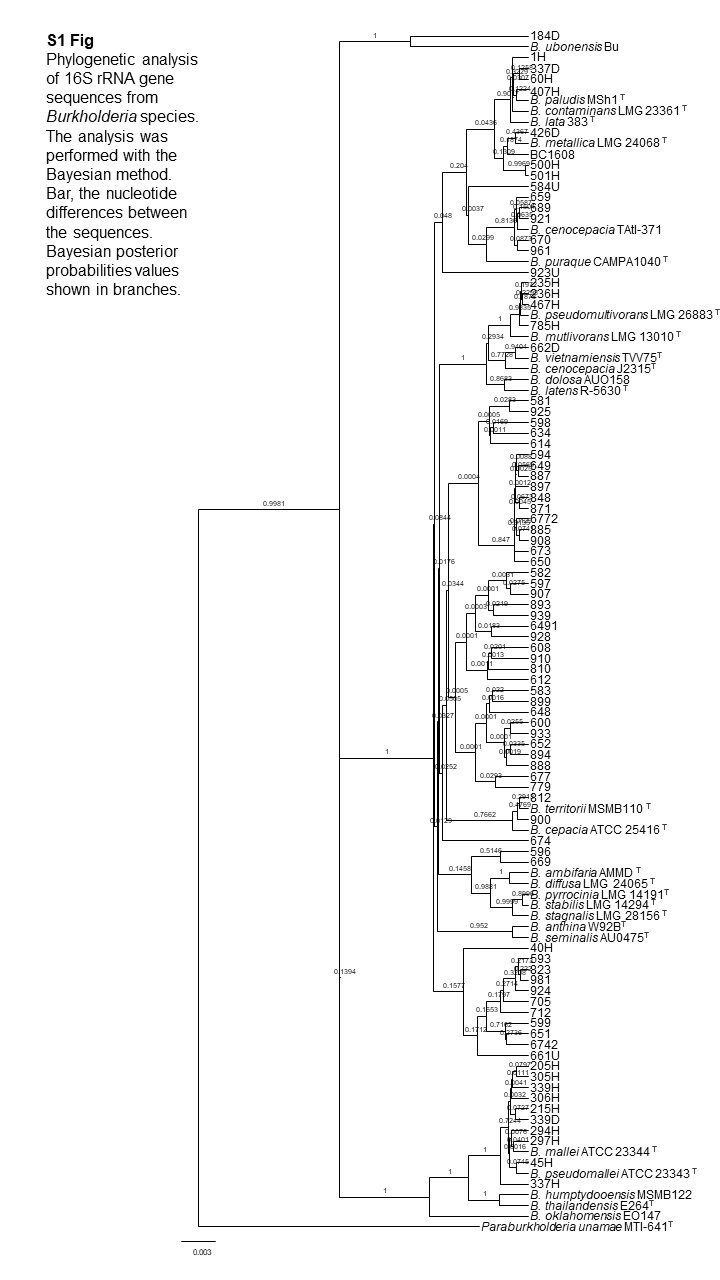

Supplement: S1 Fig — The analysis was performed with the Bayesian method. Bar, the nucleotide differences between the sequences. Bayesian posterior probabilities values shown in branches. (TIF) [file pntd.0009541.s001.tif]

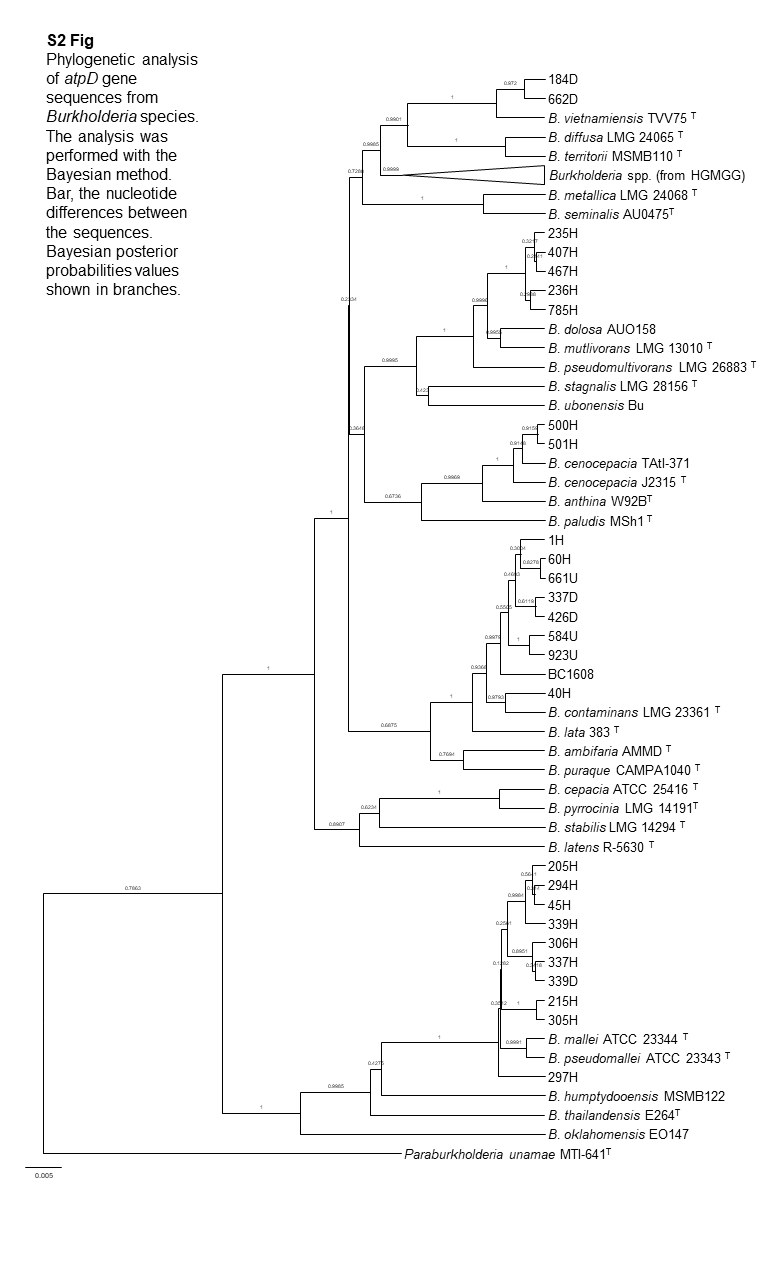

Supplement: S2 Fig — The analysis was performed with the Bayesian method. Bar, the nucleotide differences between the sequences. Bayesian posterior probabilities values shown in branches. (TIF) [file pntd.0009541.s002.tif]
