# Supplementary material for: Burkholderia species in human infections in Mexico: Identification of B. cepacia, B. contaminans, B. multivorans, B. vietnamiensis,B. pseudomallei and a new Burkholderia species
Source: PLoS Negl Trop Dis. 2021 Jun 29;15(6):e0009541. doi: 10.1371/journal.pntd.0009541 (PMC8274841; doi:10.1371/journal.pntd.0009541)
Supplement: S1 Table — (DOCX) [file pntd.0009541.s003.docx]

**Table S1. *Burkholderia* strain list isolated from patients with different ages at two hospitals in Mexico, City.**

| ***Burkholderia* species** | **Strain** | **Patient age*** | **Isolation source** |
| --- | --- | --- | --- |
| **Hospital General Dr. Manuel Gea González (HGMGG)** | | | |
| *B. cepacia* | 581  582  583  593  596  597  608  612  634  648  649  652  659  669  670  673  674  689  705  712  812  848  887  893  894  899  907  928  939  961  6772 | 29  87  60  70  58  ND  48  54  81  27  ND  39  27  47  34  28  64  1  65  72  78  1  ND  6  6  6  5  20  ND  ND  ND | Bronchus secretion culture |
|  | 888 | 5 | Hemoculture |
|  | 900 | ND | Culture from catheter |
|  | 924 | 84 | Expectoration culture |
|  | 600  677  810  823  910  933 | ND  ND  8  ND  50  ND | Bronchus secretion culture |
|  | 594  599  614  650  921  923  925  6491  6742 | 87  88  58  ND  48  62  39  ND  ND | Bronchus secretion culture |
|  | 885 | 5 | Feces culture |
|  | 897 | 52 | Cervical wound culture |
|  | 908 | 48 | Pharyngeal exudates culture |
|  | 598  871 | ND  ND | Bronchus secretion culture |
| **Hospital Infantil de México Federico Gómez (HIMFG)** | | | |
| *B. contaminans* | 40H | 1-5 | Pharyngeal exudates |
|  | 337D  1H  60H | 1-5  1-5  1-5 | Pharyngeal exudates |
|  | 426D  584U  661U  923U  BC1608 | 1-5  1-5  1-5  1-5  1-5 | Pharyngeal exudates |
|  | 407H | 1-5 | Pharyngeal exudates |
| *B. multivorans* | 235H  236H  785H  467H | 1-5  1-5  1-5  1-5 | Pharyngeal exudates |
| *B. pseudomallei* | 45H  205H  294H  297H  306H  305H  339H  337H | 1-5  1-5  1-5  1-5  1-5  1-5  1-5  1-5 | Pharyngeal exudates |
| *B. vietnamiensis* | 184D  662D | 1-5  1-5 | Pharyngeal exudates |
| *Burkholderia* sp. | 500H  501H | 1-5  1-5 | Pharyngeal exudates |

*, The exact age of children (1-5) was unavailable. ND, age not determined.
