# Supplementary material for: Burkholderia species in human infections in Mexico: Identification of B. cepacia, B. contaminans, B. multivorans, B. vietnamiensis,B. pseudomallei and a new Burkholderia species
Source: PLoS Negl Trop Dis. 2021 Jun 29;15(6):e0009541. doi: 10.1371/journal.pntd.0009541 (PMC8274841; doi:10.1371/journal.pntd.0009541)
Supplement: S2 Table — (DOCX) [file pntd.0009541.s004.docx]

**Table S2. Genomic features of *Burkholderia* strains isolated from human infections in Mexico.**

| ***Burkholderia***  **Species** | **Strain** | **Genome**  **size**  **(bp)** | **DNA**  **coding**  **(bp)** | **G+C**  **(%)** | **Scaffolds** | **Total**  **genes** | **Protein**  **coding**  **genes** | **RNA**  **genes** | **ST** | **Accession**  **number at NCBI** |
| --- | --- | --- | --- | --- | --- | --- | --- | --- | --- | --- |
| *B. cepacia* | 810 | 7528867 | 6587428 | 66.58 | 232 | 7227 | 7111 | 74 | 9 | JAEDXI000000000 |
| *B. cepacia* | 848 | 8715439 | 7604264 | 66.56 | 166 | 8261 | 8143 | 74 | 9 | JAEDXH000000000 |
| *B. cepacia* | 871 | 8888639 | 7763294 | 66.44 | 518 | 8685 | 8546 | 92 | 9 | JAEDXG000000000 |
| *B. cepacia* | 921 | 7514347 | 6575611 | 66.58 | 178 | 7181 | 7066 | 73 | 9 | JAEDXF000000000 |
| *B. contaminans* | 1H | 7426170 | 6482841 | 65.87 | 107 | 7129 | 7021 | 70 | 482 | JAEDXE000000000 |
| *B. contaminans* | 40H | 8530243 | 7457639 | 66.16 | 108 | 8026 | 7912 | 71 | 102 | JAEDXD000000000 |
| *B. contaminans* | 407H | 8418957 | 7382714 | 66.52 | 507 | 8144 | 8010 | 95 | 482 | JAEDXC000000000 |
| *B. contaminans* | 584U | 8252151 | 7230633 | 66.60 | 103 | 7668 | 7556 | 76 | 482 | JAEDXB000000000 |
| *B. contaminans* | 661U | 8260099 | 7239085 | 66.59 | 127 | 7701 | 7593 | 72 | 482 | JAEDXA000000000 |
| *B. multivorans* | 785H | 6507018 | 5711956 | 66.86 | 565 | 6436 | 6299 | 92 | 1867* | JAEDWZ000000000 |
| *B. pseudomallei* | 294H | 7217564 | 6102787 | 67.97 | 201 | 6355 | 6253 | 63 | 1872* | JAEDWY000000000 |
| *B. vietnamiensis* | 184D | 7035551 | 6096664 | 66.81 | 123 | 6573 | 6464 | 67 | 596 | JAEDWX000000000 |
| *Burkholderia* sp*.* | 500H | 7667777 | 6735765 | 66.92 | 64 | 7194 | 7078 | 72 | ND | JAENHS000000000 |

ST, Sequence Typing. *New ST in the species. ND, not determined.
